# Different populations of *Aedes aegypti* and *Aedes albopictus* (Diptera: Culicidae) from Central Africa are susceptible to Zika virus infection

**DOI:** 10.1371/journal.pntd.0008163

**Published:** 2020-03-23

**Authors:** Basile Kamgang, Marie Vazeille, Armel Tedjou, Aurélie P. Yougang, Theodel A. Wilson-Bahun, Laurence Mousson, Charles S. Wondji, Anna-Bella Failloux

**Affiliations:** 1 Centre for Research in Infectious Diseases, Department of Medical Entomology, Yaoundé, Cameroon; 2 Institut Pasteur, Department of Virology, Unit of Arboviruses and Insect Vectors, Paris, France; 3 Department of Animal Biology, Faculty of Sciences, University of Yaoundé I, Yaoundé, Cameroon; 4 Faculty of Science and Technology, Marien Ngouabi University, Brazzaville, Congo; 5 Vector Biology Department, Liverpool School of Tropical Medicine, Liverpool, United Kingdom; INDEPENDENT RESEARCHER, UNITED STATES

## Abstract

Zika virus (ZIKV) is a *Flavivirus* (*Flaviviridae*) transmitted to humans mainly by the bite of an infected *Aedes* mosquitoes. *Aedes aegypti* is the primary epidemic vector of ZIKV and *Ae*. *albopictus*, the secondary one. However, the epidemiological role of both *Aedes* species in Central Africa where *Ae*. *albopictus* was recently introduced is poorly characterized. Field-collected strains of *Ae*. *aegypti* and *Ae*. *albopictus* from different ecological settings in Central Africa were experimentally infected with a ZIKV strain isolated in West Africa. Mosquitoes were analysed at 14- and 21-days post-exposure. Both *Ae*. *aegypti* and *Ae*. *albopictus* were able to transmit ZIKV but with higher overall transmission efficiency for *Ae*. *aegypti* (57.9%) compared to *Ae*. *albopictus* (41.5%). In addition, disseminated infection and transmission rates for both *Ae*. *aegypti* and *Ae*. *albopictus* varied significantly according to the location where they were sampled from. We conclude that both *Ae*. *aegypti* and *Ae*. *albopictus* are able to transmit ZIKV and may intervene as active Zika vectors in Central Africa. These findings could contribute to a better understanding of the epidemiological transmission of ZIKV in Central Africa and develop suitable strategy to prevent major ZIKV outbreaks in this region.

## Author summary

Zika virus (ZIKV), isolated for the first time in Uganda in 1947, is transmitted to human beings mainly by the bite of an infected mosquitoes belonging to the *Aedes* genus notably *Ae*. *aegypti* and *Ae*. *albopictus*. Both *Aedes* species are present in Central Africa, however their epidemiological role is poorly characterized. Here, we assessed the ability of *Ae*. *aegypti* and *Ae*. *albopictus* collected in different ecological settings in Central Africa to transmit a ZIKV strain isolated in West Africa. Analysis showed that both *Ae*. *aegypti* and *Ae*. *albopictus* are able to transmit ZIKV but with higher overall transmission efficiency for *Ae*. *aegypti* compared to *Ae*. *albopictus*. In addition, disseminated infection and transmission rates for both *Aedes* species varied significantly according to the sampling location. Overall, our result suggests that in Central Africa, *Ae*. *aegypti* is more competent to transmit ZIKV than *Ae*. *albopictus* although parameters such as the feeding behaviour, longevity and mosquito densities can modulate pathogens transmission in nature. These findings could contribute to a better understanding of the epidemiological transmission of ZIKV in Central Africa and develop suitable strategy to prevent major ZIKV outbreaks in this region.

## Introduction

Zika virus (ZIKV) is a recently emerged, mosquito-borne virus belonging to the *Flavivirus* genus isolated initially from a sentinel monkey, at the Zika forest in Uganda in 1947 [1]. For decades, only sporadic circulation of ZIKV was documented in Africa and South East Asia [2] with two main genotypes; African and Asian genotypes [3, 4]. Nevertheless, major epidemics were reported in Micronesia in 2007 [5], the Pacific region in 2013–2014 [6–8], and Brazil in 2015, the starting point of the unprecedented outbreak affecting several countries and territories in the Americas [9]. During the same period, ZIKV transmission was reported in some African countries such as Cabo Verde [10], Guinea Bissau [11], and Angola [12]. Because of the association of ZIKV with microcephaly [13], Guillain-Barré syndrome [14], and myelitis [15], ZIKV was declared as a Public Health emergency of International concern in 2016 [16]. ZIKV can be transmitted by sexual intercourse, blood transfusion, and from mother to child (breast milk and in utero) or by physical contacts [17]. However, vertebrates including humans are mainly infected through the bite of an infected mosquitoes belonging to the *Aedes* genus. Two main transmission cycles are well documented: (i) a sylvatic cycle between non-human primates and arboreal canopy-dwelling mosquitoes including *Ae*. *africanus*, *Ae*. *furcifer*, *Ae*. *luteocaphalus*, *Ae*. *opok*, and *Ae*. *vittatus* mainly in Africa and (ii) an urban cycle between humans and domestic mosquitoes such as *Ae*. *aegypti* and *Ae*. *albopictus* [18, 19]. Both epidemic vectors, *Ae*. *aegypti* and *Ae*. *albopictus*, are well established in Africa where *Ae*. *aegypti* is native [20]. While *Ae*. *albopictus* originated from Asia, it was reported for the first time in Central Africa in early 2000 in Cameroon [21] and has progressively colonized almost all countries in the region where it tends to supplant the resident species *Ae*. *aegypti* [22–25]. Interestingly, the first ZIKV strain isolated from *Ae*. *albopictus* was in Gabon (Central Africa) in mosquitoes collected in urban areas [26], highlighting the potential role of this species as a ZIKV vector in the region. In contrast, *Ae*. *aegypti* has never been found naturally infected with ZIKV in the Central African region. However, from a study with blood donors showing that ZIKV is circulating in Cameroon, nearly 5–10% of people from six towns have been exposed to ZIKV infections [27]. The vector competence of natural *Aedes* populations from Central Africa has remained unclear. It has been demonstrated that the level of vector competence varies according to mosquito populations and ZIKV strains [28–31]. To fill this gap, we assessed the ability of *Ae*. *aegypti* and *Ae*. *albopictus* populations from Central Africa to transmit a ZIKV strain isolated in West Africa.

## Methods

### Ethics statement

This study was approved by the Cameroonian national ethics committee for human health research N°2017/05/911/CE/CNERSH/SP. Oral consent to inspect the potential breeding sites was obtained in the field from household or business occupants. The Institut Pasteur animal facility has received accreditation from the French Ministry of Agriculture to perform experiments on live animals in compliance with the French and European regulations on care and protection of laboratory animals (EC Directive 2010/63, French Law 2013–118, February 6th, 2013). All experiments were approved by the Ethics Committee #89 and registered under the reference APAFIS#6573-201606l412077987 v2.

### Mosquito collections

**Fig 1:**
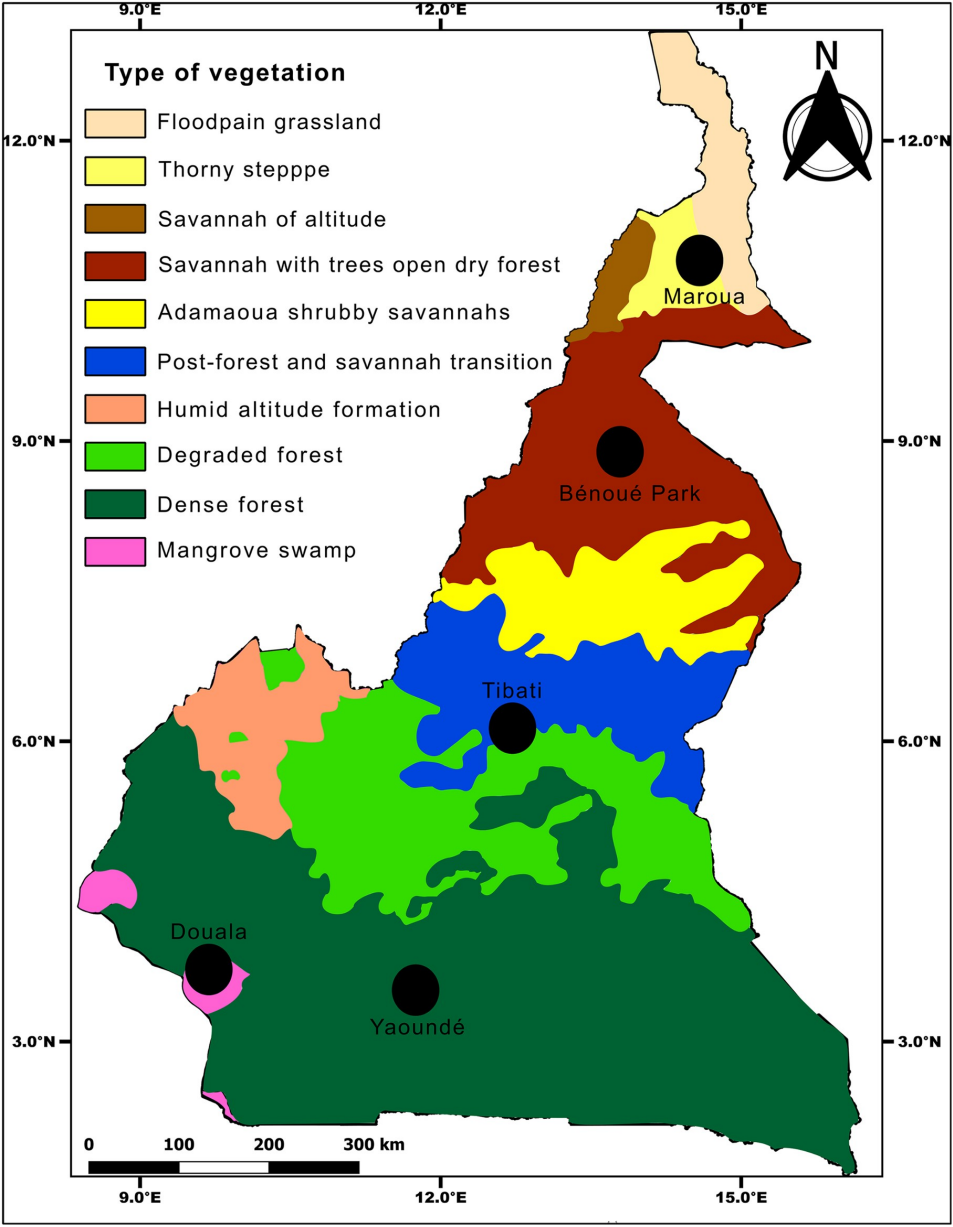
Map of Cameroon vegetation showing the sampling sites.

Mosquitoes were sampled as immature stages from August 2017 to April 2018 in several locations (Table 1) in Central Africa including Brazzaville in the Republic of Congo, and Yaoundé, Douala, Tibati, Maroua and Benoué National Park in Cameroon (Fig 1). Detailed characteristics of each collection site are presented in previous studies [22, 23, 32]. Larvae/pupae of *Aedes* mosquitoes collected from a minimum of 20 containers per site were transported to insectaries and pooled together according to the city and maintained until adults before morphological identification. Adults from same location and species were reared at 28°±1°C under 12h dark: 12h light cycle and 80% relative humidity. Eggs obtained (Table 1) were transported to the Institut Pasteur in Paris, reared to adult stage under controlled insectary conditions and used to challenge with ZIKV.

**Table 1:** Origin of *Ae*. *aegypti* and *Ae*. *albopictus* used for vector competence.

Location	Species	Generation
Yaoundé urban	*Ae*. *albopictus*	G2
Tibati	*Ae*. *albopictus*	G2
Douala	*Ae*. *albopictus*	G2
Brazzaville	*Ae*. *albopictus*	G5
Yaoundé urban	*Ae*. *aegypti*	G2
Yaoundé rural	*Ae*. *aegypti*	G2
Bénoué Parc	*Ae*. *aegypti*	G4
Brazzaville	*Ae*. *aegypti*	G2
Maroua	*Ae*. *aegypti*	G2
Douala	*Ae*. *aegypti*	G2

### Zika virus strain used

The ZIKV strain (*Ae*. *taylori*-tc/SEN/1984/41662-DAK) was isolated in December 1984 from *Ae*. *taylori* mosquito in Dakar, Senegal (GenBank accession number: KU955592) [33]. The strain was provided by EVAg (https://www.european-virus-archive.com/). The strain was passaged four times on BHK-21 cells and freeze-dried before the shipment. Upon receipt, the sample was re-suspended in 400 μL of distilled water. The viral stock for mosquito infections was prepared after two passages of the isolate on Vero CCL-81 cells (ATCC, VA, USA) maintained at 37°C. Once cytopathic effect was detected (60–72 h after infection), supernatants were collected and adjusted to 10% fetal bovine serum (Life Technologies, CA, USA), aliquoted into 1.5 mL samples, and frozen at -80°C until thawed and used to prepare the blood-virus suspensions used to expose mosquitoes to ZIKV. The viral titre was estimated by serial 10-fold dilutions on Vero cells expressed in pfu (plaque-forming units)/mL.

### Challenged of mosquitoes with ZIKV

For each population, six batches of 60, 7–10 day-old females were exposed to an infectious blood meal containing 1.4 mL of washed rabbit erythrocytes and 700 μL of viral suspension. The blood meal was supplemented with adenosine 5’-triphosphate (ATP) as a phagostimulant at a final concentration of 5 mM. The titre of infectious blood-meals provided to mosquitoes was 10^7^ pfu/mL using a Hemotek membrane feeding system (Hemotek Ltd., Blackburn, UK). Mosquitoes were allowed to feed for 20 min through a pork intestine membrane covering the base of a Hemotek feeder maintained at 37°C. Fully engorged females were sorted on wet ice, transferred to cardboard containers covered by mosquito netting, and fed *ad libitum* with 10% sucrose under controlled conditions (28±1°C, relative humidity of 80%, light: dark cycle of 12h: 12h).

### Vector competence indices

For each mosquito examined, body (abdomen and thorax), and head were tested respectively for infection and dissemination rates at 14 and 21 days post-exposure (dpe). For this purpose, each part was ground individually in 300 μL of DMEM medium (Invitrogen, CA, USA) supplemented with 2% fetal serum bovine (FBS), and centrifuged at 10,000×g for 5 min at 4°C. The supernatant was processed for viral titration as described below. Saliva was collected from individual mosquitoes using techniques of forced salivation as described previously [34]. Briefly, mosquitoes were cool anesthetized, wings and legs of each mosquito were removed, and the proboscis inserted into a plastic pipette tip of 20 μL containing 5 μL of FBS. After 30 minutes, FBS containing saliva was mixed with 45 μL of DMEM for titration.

Infection rate (IR) refers to the proportion of mosquitoes with infected body (abdomen and thorax) among tested mosquitoes. Disseminated infection rate (DIR) corresponds to the proportion of mosquitoes with infected head among the previously detected infected mosquitoes (i.e. virus-positive abdomen/thorax). Transmission rate (TR) represents the proportion of mosquitoes with infectious saliva among mosquitoes with disseminated infection. Vector competence can be summarised by the transmission efficiency (TE) which was calculated as the proportion of mosquitoes with infectious saliva among total of mosquitoes tested [28].

### Viral titration

Body and head suspensions were serially diluted and inoculated onto monolayers of Vero cells in 96-well plates. Cells were incubated for 7 days at 37°C then stained with a solution of crystal violet (0.2% in 10% formaldehyde and 20% ethanol). Presence of viral particles was assessed by detection of cytopathic effect (CPE). Saliva was titrated on monolayers of Vero cells in 6-well plates incubated 7 days under an agarose overlay. Saliva titres were expressed as pfu/saliva.

### Statistical analysis

All statistical analyses were performed with R software. Qualitative variables were expressed as proportion and compared using Fisher’s exact test and quantitative variables by mean and compared using non-parametric test of Kruskal-Wallis because of non-normal distribution. Pairwise comparisons were performed using Fisher’s exact test for proportions and Kruskal-Wallis test for means. For multiple comparisons, the Bonferroni correction was applied. *P-value* <0.05 was considered as statistically different.

## Results

### Infection and dissemination rates in *Ae*. *albopictus* and *Ae*. *aegypti*

**Fig 2:**
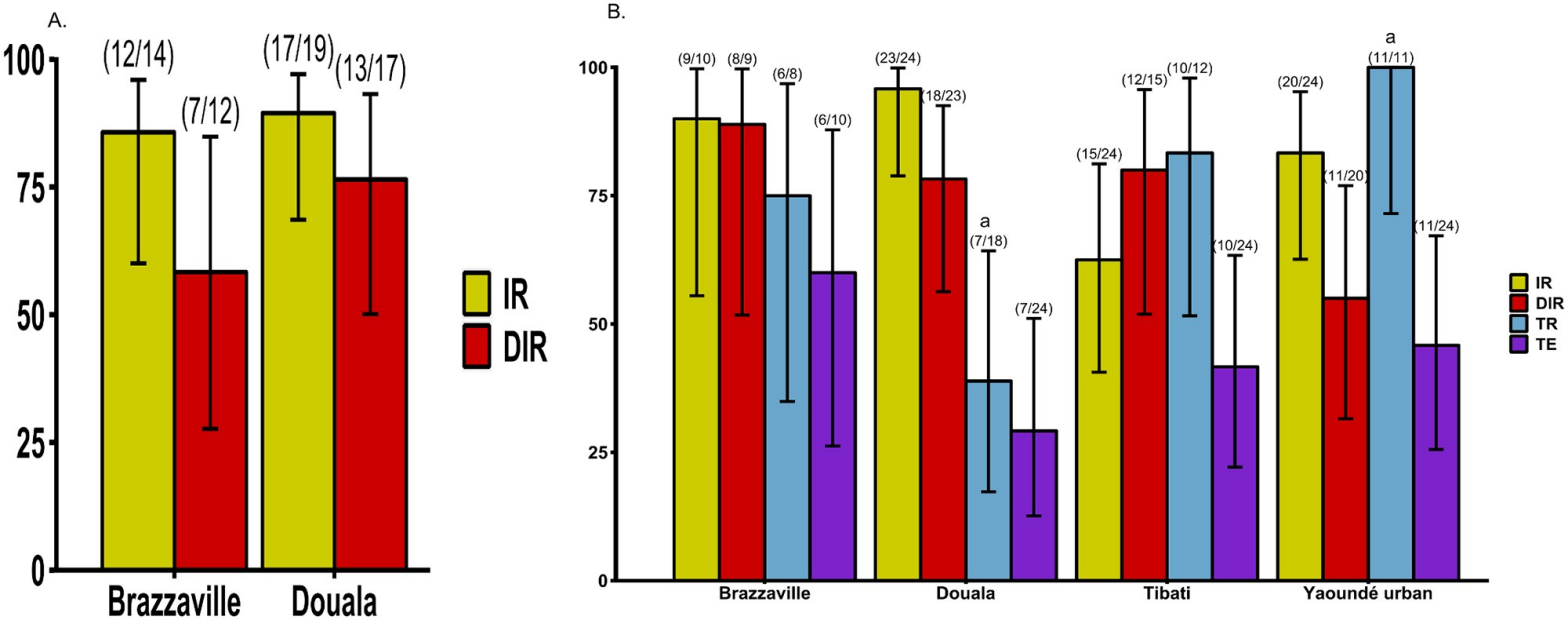
Infection, dissemination, transmission rates and transmission efficiency of *Ae*. *albopictus* from Central Africa. A) Infection and dissemination rates at 14 days post-exposure (dpe). B) Infection, dissemination, transmission rates and transmission efficiency at 21 dpe. Error bars show the 95% confidence interval. In brackets, the number of mosquitoes examined. IR: the proportion of mosquitoes with infected body among engorged mosquitoes; DIR: the proportion of mosquitoes with infected head among mosquitoes with infected body; TR: the proportion of mosquitoes with infectious saliva among mosquitoes with infected head. The lowercase letter on the top of some indices indicates the significant difference for pairwise comparisons. When the same letter is shared by several populations, this indicates that the difference is significant (*P*<0.05) between them.

**Fig 3:**
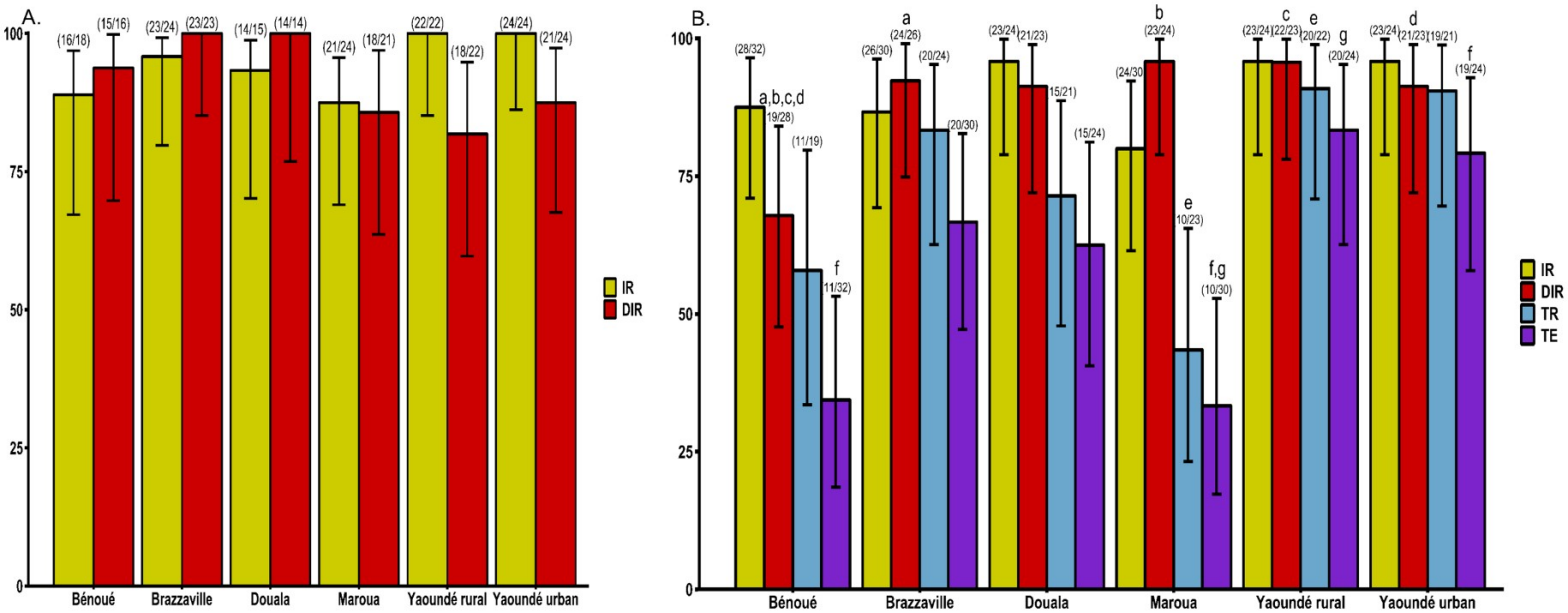
Infection, dissemination, transmission rates and transmission efficiency of *Ae*. *aegypti* from Central Africa. A) Infection and dissemination rates at 14 days post-exposure (dpe). B) Infection, dissemination, transmission rates and transmission efficiency at 21 dpe. Error bars show the 95% confidence interval. In brackets, the number of mosquitoes examined. IR: the proportion of mosquitoes with infected body among engorged mosquitoes; DIR: the proportion of mosquitoes with infected head among mosquitoes with infected body; TR: the proportion of mosquitoes with infectious saliva among mosquitoes with infected head. The lowercase letter on the top of some indices indicates the significant difference for pairwise comparisons. When the same letter is shared by several populations, this indicates that the difference is significant (*P*<0.05) between them.

To determine whether *Ae*. *aegypti* (six populations) or *Ae*. *albopictus* (four populations) were more likely to sustain ZIKV outbreak in Central Africa, the ability of the virus to replicate and disseminate were examined at 14 and 21 dpe as well as ZIKV particles secreted in saliva (only at 21 dpe) (Figs 2 and 3).

When tested at 14 dpe, both the Douala and Brazzaville populations of *Ae*. *albopictus* were highly susceptible to infection and dissemination with ZIKV (Fig 2A), and at 21 dpe, all four populations were susceptible. However, the Douala (96%) population was significantly more susceptible (Fisher’s exact test: *P* = 0.02) than the one from Tibati (63%) (Fig 2B). Dissemination rates ranged from 55% for Yaoundé to 89% for Brazzaville populations (Fig 2B), but these rates were not significantly different (Fisher’s exact test: *P* = 0.11).

For *Ae*. *aegypti*, no significant variation was found for infection rate (IR) and disseminated infection rate (DIR) at 14 dpe (Fig 3A). Meanwhile, at 21 dpe, DIR varied significantly (Fisher’s exact test: *P* = 0.02) from 76% to 90% according to the population origin; however, no significant variation was reported for IR (Fig 3B). Pairwise comparisons for DIR revealed significant difference (Fisher’s exact test: *P* < 0.04) between Bénoué population and all other populations tested except Douala and Yaoundé urban populations. Overall, while IR were similar (Fisher’s exact test: *P* > 0.107) for *Ae*. *aegypti* (94 and 90%) and *Ae*. *albopictus* (88 and 82%) when tested at 14 or 21 dpe, respectively, DIR was significantly higher (Fisher’s exact test: *P* < 0.009) in *Ae*. *aegypti* (91% and 88.4%) than in *Ae*. *albopictus* (69 and 73%) when tested at 14 or 21 dpe, respectively.

### Transmission rate and efficiency

**Fig 4:**
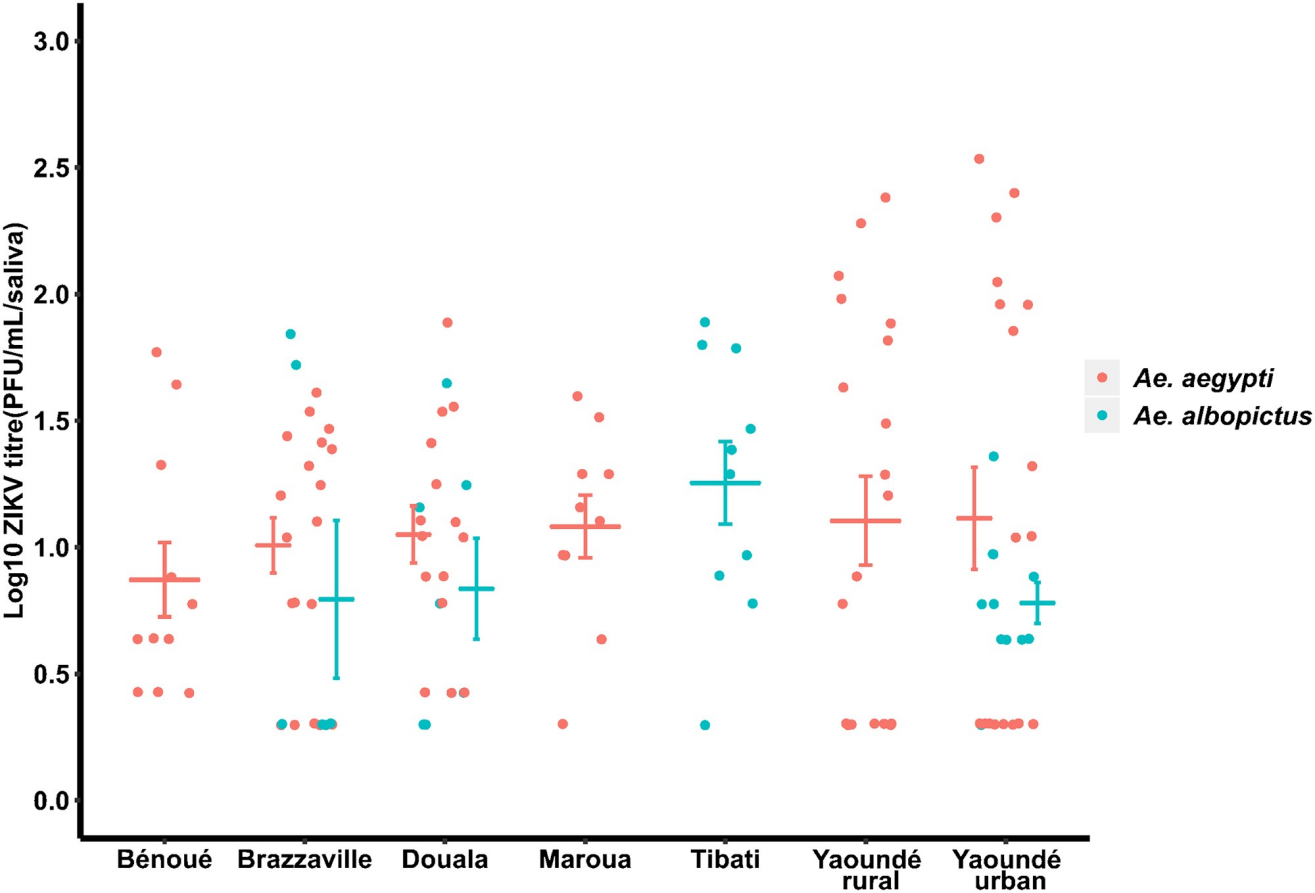
Zika virus titres in saliva of *Ae*. *aegypti* and *Ae*. *albopictus* at 21 days post-exposure. The bars indicate the confidence interval of the mean for viral load in each population.

In *Ae*. *albopictus*, transmission rate (TR) and transmission efficiency (TE) varied according to the population tested (Fig 2B). For the TR, pairwise comparisons revealed a significant difference (Fisher’s exact test: *P* = 0.007) between Douala (39%) population and Yaoundé urban (100%) population; however, for other comparisons no significant difference (Fisher’s exact test: *P* > 0.05) was reported. While, for the TE no significant difference (Fisher’s exact test: *P* = 0.37) (Figs 2B and 3B) was reported between populations after pairwise comparisons. Similarly, in *Ae*. *aegypti*, TR varied significantly (Fisher’s exact test: *P* = 0.0014) according to the population with lowest TR reported in two populations from northern part of Cameroon, Bénoué (56%) and Maroua (43%) (Fig 3B). Pairwise comparisons showed a significant difference (Fisher’s exact test: *P* < 0.021) between Maroua and Yaoundé (urban and rural) populations. Contrary to *Ae*. *albopictus*, the TE for *Ae*. *aegypti* was significantly different (Fisher’s exact test: *P* < 0.0001) according to the population origin. Pairwise comparisons indicated significant differences (Fisher’s exact test: *P* < 0.01) between Bénoué population and both Yaoundé populations (urban and rural), and between Maroua and both Yaoundé populations also. When all populations of each species were analysed together, *Ae*. *aegypti* (56%) exhibited higher TEs than *Ae*. *albopictus* (42%) (Fisher’s exact test: *P* = 0.02). Overall, ZIKV titres were significantly higher in *Ae*. *aegypti* compared to *Ae*. *albopictus* (Chi-squared test: χ^2^ = 6.4527, df = 1, *P* = 0.01). In *Ae*. *aegypti*, significant differences in viral loads were reported according to population (Chi-squared test: χ^2^ = 21.406, df = 5, *P* = 0.01) with lowest titres in Maroua population, and highest in Brazzaville population. While in *Ae*. *albopictus*, no significant variation of ZIKV titres was observed (Chi-squared test: χ^2^ = 2.65, df = 3, *P* = 0.44) (Fig 4).

## Discussion

The first evidence of ZIKV circulation was reported in Africa [1]. In Central Africa, ZIKV circulation in human populations was confirmed in Cameroon, Central African Republic (CAR), and Gabon [18]. As an example, in Cameroon, 2 to 10% blood donors were ZIKV-positive [27]. In Central Africa, exposure to ZIKV has also been confirmed in animals, monkeys and bats [18], and ZIKV was detected in two mosquito species in CAR (*Ae*. *africanus* and *Ae*. *opok*) [35], and *Ae*. *albopictus* in Gabon in 2007 [26]. Up to now, no data on vector competence to ZIKV is available for mosquitoes from Central Africa.

In this study, we assessed for the first time the ability of *Ae*. *aegypti* and *Ae*. *albopictus* collected in different ecological settings in Central Africa to transmit a ZIKV strain isolated from sylvatic mosquitoes, *Ae*. *africanus*, collected in Dakar in 1984. We demonstrated that this ZIKV strain was able to replicate, disseminate, and be secreted in saliva of both *Ae*. *aegypti* and *Ae*. *albopictus*. The results of these experiments indicate that ZIKV could be transmitted during blood feeding. Our analysis showed high infection, dissemination, and transmission rates in both species which is in agreement with previous experiments using ZIKV from the African lineage [36–39]. Disseminated infection and transmission rates varied significantly according to *Ae*. *aegypti* and *Ae*. *albopictus* populations. This result is consistent with previous studies showing the level of infection varied among mosquito populations [28, 30]. Overall, transmission efficiency and ZIKV titre in saliva were significantly higher in *Ae*. *aegypti* than in *Ae*. *albopictus* corroborating the main role of *Ae*. *aegypti* in ZIKV transmission compared to *Ae*. *albopictus* [40, 41]. Nevertheless, lower transmission rates and saliva ZIKV titres in *Ae*. *aegypti* were found for two populations from northern part of Cameroon, Benoué and Maroua, probably due to the presence of specific refractory genes [42, 43]. Beside the mosquito genetic background, mosquito microbiome can modulate arbovirus transmission [44–46]. In addition, refractoriness of mosquito to ZIKV can also be caused by mosquito immune responses since it was demonstrated that anti-viral immunity in mosquito vectors is critical to prevent virus replication and transmission [47].

Likewise, *Ae*. *aegypti* alone was reported in the northern part of Cameroon whereas both *Ae*. *aegypti* and *Ae*. *albopictus* were found sympatric in the southern part. The invasive species, *Ae*. *albopictus*, first detected in Cameroon in 2000 [21], tends to replace the native species, *Ae*. *aegypti*. However, both are suspected to contribute to ZIKV transmission. Interestingly, *Ae*. *albopictus* collected in Gabon (Central Africa) in 2007 during concurrent chikungunya/dengue outbreak was found naturally infected with ZIKV [26]. Level of transmission rates reported in both *Aedes* species suggested that both species can potentially cause major outbreaks in the region. However, it is important to highlight that other parameters such as the feeding behaviour and mosquito densities can modulate pathogens transmission in nature. Indeed, preliminary studies in Central Africa showed that *Ae*. *aegypti* occurs across the region and *Ae*. *albopictus* is found under 6°N latitude. Meanwhile, in the sympatric areas, *Ae*. *albopictus* is almost dominant except in some rare locations [22–24], and irrespective to the season in the areas with short dry season [48]. It was also demonstrated that in Yaoundé (Cameroon), *Ae*. *albopictus* preferentially fed on humans rather than on available domestic animals. In this study, mixed blood meals animal-human were detected, confirming that this species could act also as a bridge vector for zoonotic pathogens [49]. In Central Africa, data on blood meal preference and biting behavior of *Ae*. *aegypti* are quite scarce. Further studies in this regard, could help to determine the epidemiological role of each species in ZIKV transmission. In addition, as our results showed that the transmission rate in both species vary according to the population origin, mosquito microbiome composition, genetic structure and gene flow of both species across the central African region, should be further explored to determine their impact on ZIKV transmission. These findings highlight the urgent need to plan a vector surveillance program and control methods against Zika vectors in the region in order to prevent future outbreaks.
